# Optimizing Enzymatic Pretreatment of Wet-Grade Maize Distiller’s Dried Grains with Solubles and Maize Germ Meal for Enhanced Metabolizable Energy Utilization in Broilers

**DOI:** 10.3390/ani15192819

**Published:** 2025-09-26

**Authors:** Mengli Zheng, Huixin Zhang, Jing An, Haoran Wei, Tieying Zhang, Qinghua Chen

**Affiliations:** 1 College of Animal Science and Technology, Hunan Agricultural University, Changsha 410128, China; 2 State Key Laboratory of Animal Nutrition, Institute of Animal Science, Chinese Academy of Agricultural Sciences, Beijing 100193, China; 3 College of Food Science and Engineering, Qingdao Agricultural University, Qingdao 266109, China

**Keywords:** maize DDGS, maize germ meal, solid-state fermentation, the metabolic energy, broilers

## Abstract

This study addressed the challenges posed by wet-grade maize distiller’s dried grains with solubles (DDGS), which are characterized by high moisture and complex fibers that limit their storage and utilization in poultry feed. Three experiments were conducted to enhance their nutritional value through enzymatic and solid-state fermentation treatments. In vitro pre-digestion using multiple enzymes significantly improved dry matter solubility (DMS) and reducing sugar yield for maize DDGS and the ingredient maize germ meal (MGM). Using optimized parameters, wet-based DDGS-MGM was subjected to solid-state fermentation with 500 mg/kg of cellulase and 200 mg/kg of the X1 enzyme (a laboratory-developed multi-enzyme complex), and this treatment enhanced both DMS and reducing sugar yield, and the resulting fermented product was subsequently applied in further experiments. In the broiler trial, forty 22-day-old Arbor Acres broilers with similar body weights were randomly assigned to five treatment groups, including the control group, (50% DDGS + 50% MGM) unfermented group, (62.5% DDGS + 37.5% MGM) unfermented group, (50% DDGS + 50% MGM) fermented group, and (62.5% DDGS + 37.5% MGM) fermented group, with eight replicates per treatment (one broiler per replicate). Replacement of 30% of the basal diet with fermented 50:50 DDGS-MGM material significantly increased apparent metabolizable energy (AME) and nitrogen-corrected AME by 2.74 MJ/kg and 2.73 MJ/kg, respectively, corresponding to improvements of 39.60% and 40.81% compared to the unfermented control (*p* < 0.05). Economic analysis indicated that using 5% fermented DDGS-MGM in feed reduced cost by 20.45 RMB per metric ton. These findings demonstrate that bioprocessing can improve the utilization and economic value of maize processing by-products, although further validation under practical conditions is needed.

## Simple Summary

Wet-grade maize distiller’s dried grains with solubles (DDGS), characterized by high moisture content, poor storability, and elevated fiber content, exhibit low nutrient digestibility. Studies have demonstrated that enzymatic–bacterial synergistic solid-state fermentation of wet-grade maize DDGS combined with supplementary substrates effectively improved dry matter solubility and enhanced metabolizable energy (ME). To address the challenges of high moisture and complex fibrous structure in wet-grade DDGS and leverage the water-holding capacity and porous properties of maize germ meal (MGM) for synergistic fermentation, this study screened optimal enzyme combinations and dosages through in vitro pre-digestion. The effects of fermented products on ME in broilers were further evaluated. Results indicated that co-fermentation with cellulase and the X1 enzyme (xylanase) significantly increased dry matter degradation and elevated ME in the wet-grade DDGS-MGM substrate.

## 1. Introduction

The poultry industry has long faced ongoing challenges in the supply of energy and protein feeds, an issue further compounded by fluctuations in farming demand and volatility in feed prices in domestic and international markets. Consequently, identifying sustainable alternative ingredients to reduce reliance on conventional maize and soybean meal has become an urgent priority for researchers and industry stakeholders. At the same time, enhancing the utilization efficiency and economic value of maize processing by-products is increasingly recognized as a viable strategy to alleviate supply constraints.

Among various alternative ingredients, maize processing by-products such as maize germ meal (MGM), maize gluten meal, and distillers dried grains with solubles (DDGS) demonstrate considerable potential in poultry feed [1]. These materials are characterized by high nutritional density, with significantly elevated levels of protein and energy compared to unprocessed grains. In particular, maize DDGS production achieved substantial industrial scale; for instance, China’s annual output exceeded 6.87 million tons in 2024, reflecting its growing importance in the feed sector. DDGS was derived from the bioethanol production process, which involves grinding, fermentation, centrifugation, distillation, and drying, resulting in a nutrient-concentrated product with approximately three times the protein, fat, and fiber content of raw maize.

However, the broader application of maize DDGS and MGM in animal feeds has been limited by their high content of poorly digestible fiber. DDGS contain approximately 25% non-starch polysaccharides (NSPs), comprising cellulose, hemicellulose, and soluble/insoluble fiber fractions. More critically, these components form a complex matrix where cellulose, hemicellulose, and lignin are cross-linked via ferulic acid ester and ether bonds, strongly resisting enzymatic degradation. Monogastric animals such as broilers lack the endogenous enzymes needed to break down these structures—particularly lignin—resulting in low nutrient digestibility and availability. Studies show that the inclusion of DDGS exceeding 200 g/kg in broiler diets reduces weight gain, while the high crude fiber content in MGM (over four times that of whole maize) significantly depresses dry matter digestibility at elevated dietary levels [2]. These limitations underscore the necessity of developing efficient pretreatment strategies to disrupt the fiber matrix and enhance the functional quality of these feed ingredients.

Enzymatic hydrolysis and solid-state fermentation (SSF) proves effective in modifying the fiber structure of maize DDGS, thereby improving energy utilization efficiency, nutrient digestibility, and absorption in broilers. The recalcitrant fiber network in maize DDGS and MGM—composed of cellulose, hemicellulose, lignin, pectin, and ferulic acid—requires synergistic enzymatic pretreatment. Cellulase broke down cellulose to glucose. Ferulic acid esterase hydrolyzes ester bonds between ferulic acid/arabinoxylan and ferulic acid/lignin, disrupting cell wall integrity. Laccase oxidized phenolic groups in lignin lead to depolymerization, and pectinase cleaves α-1,4 glycosidic bonds in pectin, exposing the polysaccharide backbone [3,4,5,6]. Based on compositional analyses (e.g., glucose, xylose, arabinose, ferulic acid, lignin) [7,8,9], a combination of cellulase, pectinase, laccase, and ferulic acid esterase was identified as highly effective in enhancing DMS and increasing reducing sugar release. For instance, the enzyme complex X1—derived from *Aspergillus niger* X1 (isolated from waste materials in our laboratory) and exhibiting predominant xylanase activity along with xylosidase, cellulase, glucanase, glucosidase, mannanase, and pectinase activities—significantly improved the degradation of native fiber in DDGS and MGM. In our preliminary experiments, supplementation with 200 mg/kg of X1 increased the DMS rate to 10.9% and 15.58% for the insoluble fiber of maize DDGS and MGM, respectively, within 24 h. Therefore, the enzymatic hydrolysis of maize DDGS and MGM by the X1 enzyme was also carried out.

Additionally, multi-enzyme complexes supplemented in maize–soybean–DDGS broiler diets were shown to improve body weight, feed conversion ratio, and digestibility of dry matter, crude protein, and hemicellulose [10,11]. Similarly, SSF using *fungi* such as *Trichoderma reesei* or *Aspergillus niger* considerably enhanced in vitro dry matter digestibility (by 22~43%) and reduced fiber content in DDGS [12,13,14,15]. Fermented maize DDGS also increased the average daily feed intake and average daily gain in broilers [16]. These processing technologies not only provided a scientific basis for the efficient use of maize DDGS but also offered a viable approach to reducing feed costs and improving production efficiency in livestock farming. Given the nutritional value, availability, and processing costs associated especially with wet maize DDGS, optimizing SSF protocols and selecting suitable excipients remained essential research priorities.

The optimization of enzyme-microorganism combinations and processing parameters was aimed at developing a pretreatment protocol for wet-based maize DDGS directly at its production site. This approach targeted the core challenges of wet maize DDGS, such as high energy consumption, difficult storage, and costly transportation. Through low-cost pretreatment, we sought to enhance the nutritional value of both wet maize DDGS as the primary material and MGM as a supplementary ingredient—an objective relevant to both distilleries and the feed industry. Although nutritional variability in maize DDGS often arose from differences in manufacturers, processing techniques, and batch conditions, the wet-based DDGS used in this study exhibited relatively stable composition, as it had not undergone drying and was certified by the supplier. Our SSF parameters were optimized based on this consistently stable wet material. Nevertheless, future validation using samples from broader geographical sources remains necessary to confirm the general applicability of these parameters. In addition to pretreating mixtures of wet maize DDGS and MGM, we also applied the optimized parameters—using the same enzyme types with adjusted dosage—to blends of wet maize DDGS and wheat bran. The positive outcomes confirmed that the pretreatment strategy was effective across different substrates, suggesting its potential applicability to other maize processing by-products. Cost efficiency was a critical consideration in evaluating feed pretreatment technologies. Our selection of cellulase and the X1 enzyme complex was driven not only by their efficacy in improving dry matter degradation but also by the need to minimize enzymatic costs, ensuring the economic feasibility of the process for industrial adoption.

Although considerable progress has been made in improving the nutritional value of maize DDGS and MGM, several key issues remain unresolved, including the selection of efficient enzymes, the optimization of process parameters, and the economic feasibility of treatment strategies. Therefore, this study aimed to systematically evaluate the efficacy of enzymatically enhanced SSF pretreatment in enhancing the nutritional value of maize DDGS and MGM. It was hypothesized that the targeted biological processing of SSF could significantly improve the metabolizable energy (ME) and overall feeding value of diets containing these by-products. Both wet-based maize DDGS and dry-based MGM were utilized as substrates for enzymatic treatment and SSF. By establishing efficient and low-cost pretreatment parameters, this research sought to promote the sustainable and large-scale application of maize processing by-products, thereby reducing dependence on conventional feed materials and supporting the development of resource-efficient poultry production systems. Nevertheless, the practical applicability and economic viability of this technology still require further validation under commercial production conditions.

## 2. Materials and Methods

The experiment was conducted in the State Key Laboratory of Animal Nutrition (Beijing, China). The experimental protocols used in the current study were reviewed and approved by the Animal Care and Use Committee of Institute of Animal Science, Chinese Academy of Agricultural Sciences (ISA2024-185).

### 2.1. Sources of Feed, Enzyme Preparation, and Bacterial Culture

In this study, wet maize DDGS (10.37% dry matter content) and MGM (90.40% dry matter content) were purchased from a feed company in Chifeng city (China). Cellulase (from *Pichia pastoris*, 15,000 U/g of cellulase enzyme activity and 350.00 U/g of xylanase), laccase (from *white rot fungi*, 2000 U/g of enzyme activity, 52.0 U/g of cellulase, and 97.50 U/g of xylanase), and the X1 enzyme were produced by fermentation in our laboratory (Beijing, China). The X1 enzyme had a variety of enzyme activities (from *Aspergillus niger X1*, 5000 U g of xylanase, 50 U/g of xylosidase, 100 U/g of endoglucanase, 150 U/g of glucosidase, 300 U/g of pectinase, etc.), mainly xylanase activity. Ferulic acid esterase (EC 3.1.1.73, 10,000 U/g and 45 U/g of cellulase enzyme activity) and pectinase (EC 3.2.1.15, 3000 U/g of pectinase activity and no other enzyme activity) were purchased from Jinan Baisijie Bioengineering Co., Ltd. (Jinan, China). All enzyme activities represent experimentally determined values.

The process of producing cellulase by *Pichia pastoris* in our laboratory is described in the reference of Yuan et al. [17]. The X1 enzyme was an enzyme produced by *Aspergillus niger X1* SSF in our laboratory. SSF medium preparation and culture conditions for *Aspergillus niger* are as follows. Medium was prepared in glass conical flasks by mixing 7 g of wheat bran, 3 g of maize cob powder, 0.2 g of (NH_4_)_2_SO_4_, 0.1 g of KH_2_PO_4_, and 14 mL of deionized water (substrate-to-water ratio, 1:1.6, *w*/*v*). The medium was sterilized at 121 °C for 40 min. After sterilization, it was inoculated with 2 × 10^7^ spores/mL of *Aspergillus niger* spore suspension. Fermentation was carried out at 30 °C in an aerated incubator shaker at 150 rpm for 72 h. The fermented material was dried in a 45 °C oven, pulverized, and stored at 4 °C. Laccase was produced by large-scale SSF of potato dextrose agar medium inoculated with *white rot fungus L.* in the laboratory for 4 days, and the enzyme activity could be maintained at 2000 U/g. The specific fermentation process of laccase is shown in the reference of Li et al. [18]. In this experiment, the liquid-fermented seed culture was inoculated onto sterilized wheat bran medium for large-scale fermentation over 4 days. The fermented product was subsequently crushed and stored at 4 °C until use. And during pretreatment of insoluble fiber from maize DDGS or MGM, enzyme extraction was performed. The supernatant was collected by centrifugation at 6790× *g* for 5 min prior to application.

The *lactic acid bacteria* shake flask culture procedure was conducted as follows: *Lactobacillus plantarum* HX-31, obtained through UV mutagenesis screening in the laboratory, was inoculated into sterilized De Man, Rogosa, and Sharpe (MRS) medium and cultured at 37 °C with shaking at 170 r/min for 24 h for subsequent use. The MRS medium formulation (per 250 mL) included 1.25 g of ammonium citrate dibasic, 0.0625 g of manganese sulfate, 2.5 g of sodium acetate, 0.5 g of dipotassium phosphate, 10 g of glucose, and 7.5 g of yeast extract.

Cellulase activity was assayed according to the standard of NY/T 912-2020 [19]. Xylanase activity was determined following the standard of GB/T 23874-2009 [20]. Feruloyl esterase activity was measured using the method described by Al-Shwafy et al. [21]. Pectinase activity analysis adhered to the standard of QB 1502-1992 [22]. Laccase activity was quantified per Li Cailian’s procedure [18]. Xylosidase activity assessment employed Tian Haiyuan’s protocol [23]. Endoglucanase activity was evaluated based on Zhang Yiling’s methodology [24]. β-Glucosidase activity was analyzed according to Xiao Zhenlun’s approach [25].

### 2.2. Separation of Insoluble Fiber from Maize DDGS and MGM

This work employed available enzymes such as cellulase, ferulic acid esterase, laccase, pectinase, and the X1 enzyme. Since starch, fiber, and protein in maize DDGS and MGM were intertwined, the use of cellulase and other enzymes in this process might affect the full effect of enzymes and substrates and weaken the pretreatment effect of cellulase. Thus, the maize DDGS and MGM underwent a progressive processing process involving degreasing with petroleum ether according to the standard of GB/T 6433-2006 [26], starch elimination with lipase treatment according to the standard of GB/T 20806-2022 [27], and protein extraction utilizing proteases [28]. Subsequent to these procedures, the samples were rinsed thrice with deionized water to remove soluble sugars and then dehydrated at 40 °C. The resultant residues, consisting of insoluble fibers from maize DDGS and MGM, were utilized as substrates in subsequent experiments. In this process, the extraction rate of insoluble fiber from maize DDGS and MGM was about 30%, and we produced 3 kg of maize DDGS and MGM insoluble fibers for enzyme screening and optimization.

### 2.3. Single-Factor Optimization of the Effect of Four Enzyme–Substrate Ratios on Insoluble Fibers in Two Raw Materials

#### 2.3.1. Optimization of Cellulase Enzyme–Substrate Ratio

In this experiment, 2.00 g of sterilized insoluble fibers from the two source materials were measured and placed into a 50 mL sterilized plastic centrifuge tube. Acetic acid–sodium acetate buffer (pH 5.5) was used to obtain a final volume of 20 mL. Following comprehensive mixing, diverse enzyme-to-substrate ratios of cellulase (0, 250, 500, 750, 1000, or 1250 mg/kg), laccase (200 mg/kg), ferulic acid esterase (200 mg/kg), and pectinase (200 mg/kg) were administered to the experimental groups, whereas the control groups were allocated equivalent volumes of inactivated enzyme solutions (using the boiling water bath to inactivate the enzyme for 5 min, and follow-up tests were in accordance with this method of enzyme inactivation). Subsequently, 20 μL of 10 mg/mL of chloramphenicol was introduced into each tube, followed by comprehensive mixing to inhibit the growth of *bacteria* and *fungi*. The samples were subsequently incubated in an air shaker at 37 °C and 150 rpm for 24 h. Following enzymatic hydrolysis, the reaction was halted by immersing the tubes in an ice bath for 10 min to inactivate the enzymes.

Subsequent to enzyme inactivation, the samples were subjected to centrifugation at 6790× *g* for 5 min. A fraction of the supernatant was obtained for assessing reducing sugar concentration, while the residual supernatant was examined for monosaccharide composition via liquid chromatography. Subsequent to the removal of the supernatant, the precipitate was subjected to three washes with deionized water, with centrifugation at 6790× *g* for 5 min conducted after each wash to eliminate the supernatant. The washed precipitate was subsequently dried in an oven at 105 °C, and the dry matter content was quantified to determine the DMS following enzymatic hydrolysis. Three independent replicates were performed.

#### 2.3.2. Optimized Ratio of Laccase Substrates

During optimization of laccase pretreatment for insoluble fibers in maize DDGS or MGM, experimental groups received varying laccase dosages (0, 200, 300, 400, 500, or 600 mg/kg) alongside two auxiliary enzymes at 1000 mg/kg cellulase–substrate ratios. And ferulic acid esterase (200 mg/kg) and pectinase (200 mg/kg) were administered to the experimental groups. Control groups were treated with corresponding heat-inactivated enzyme solutions, following identical experimental procedures, as outlined in Section 2.3.1.

#### 2.3.3. Optimization of the Basal Ratio of Ferulic Acid Esterase Enzyme

For feruloyl esterase efficacy evaluation during pretreatment of maize DDGS or MGM insoluble fibers, experimental groups received varying feruloyl esterase dosages (0, 200, 600, 1000, 1400, or 1800 mg/kg) supplemented with 200 mg/kg of pectinase, while maintaining 1000 mg/kg of cellulase and 200 mg/kg of laccase. Control groups were administered corresponding heat-inactivated enzyme solutions, following identical experimental procedures, as outlined in Section 2.3.1.

#### 2.3.4. Optimized Ratio of Pectinase to Substrate

In the optimization of pectinase for the pretreatment of insoluble fibers in maize DDGS or MGM, experimental groups received varying pectinase dosages (0, 200, 400, 600, 800, or 1000 mg/kg), while maintaining 1000 mg/kg of cellulase, 200 mg/kg of laccase, and 200 or 600 mg/kg of feruloyl esterase (200 mg/kg of feruloyl esterase for maize DDGS and 600 mg/kg of feruloyl esterase for MGM insoluble fibers). Control groups were treated with corresponding heat-inactivated enzyme solutions. All procedures followed the protocol described in Section 2.3.1.

### 2.4. The Effect of the X1 Enzyme Combined with Cellulase on Insoluble Fiber in Two Kinds of Raw Materials

In this experiment, 2.00 g of insoluble fibers from maize DDGS and MGM were mixed with 18 mL of acetate–sodium acetate buffer (pH 5.5) in 50 mL centrifuge tubes. Test groups received 500 mg/kg of cellulase and varying X1 enzyme–substrate ratios (0, 200, 400, 600, 800, or 1000 mg/kg), while controls were treated with heat-inactivated enzyme solutions. After adding 20 μL of 10 mg/mL of chloramphenicol, the mixtures were homogenized and incubated at 37 °C with 150 rpm agitation in an orbital shaker for 24 h. Enzymatic reactions were terminated by ice bath incubation for 10 min, followed by centrifugation at 6790× *g* for 5 min. Supernatants were analyzed for the reducing sugar content and monosaccharide composition via liquid chromatography. Pellets were washed with deionized water and re-centrifuged. All procedures followed the protocol described in Section 2.3.1. Three independent replicates were performed.

### 2.5. Optimization of SSF Conditions of Maize DDGS-MGM

A 100 g dry matter basis mixture was prepared by combining 167.03 g of wet basis maize DDGS and 55.19 g of air-dried MGM (mixed at a 50:50 ratio on a dry matter basis, 50% DDGS + 50% MGM), and then inoculated with laboratory-preserved *Lactobacillus plantarum* (>10^8^ CFU/mL, inoculum size 5%), and SSF enzyme preparations (0, 15, 30, 45, 60, 75, or 90 mg/kg on a dry matter basis) of the X1 enzyme and (0, 15, 45, 75, 105, 135, or 165 mg/kg on a dry matter basis) cellulase. The mixed samples were mixed with the appropriate amount of enzymes and bacteria (the specific addition amount was based on the subsequent single-factor test results), and then placed in three 50 mL centrifuge tubes with samples after sterilization and placed in an incubator for anaerobic fermentation (fermentation conditions at 37 °C; moisture was 55%; the fermentation time was 48 h). After the fermentation, it was placed in an ice bath for 10 min to inactivate the enzyme. After mixing the fermented samples, 2.00 g of the sample was taken into a 10 mL centrifuge tube, and an appropriate amount of deionized water was added to fully mix. After centrifugation at 6790× *g* for 5 min, the supernatant was taken to determine the reducing sugar content and monosaccharide content in the sample. In addition, after the supernatant was discarded, the precipitate was washed with deionized water, and the supernatant was discarded after centrifugation. All procedures followed the protocol described in Section 2.3.1. This experiment was repeated three times.

#### 2.5.1. Single-Factor Optimization of Cellulase Enzyme Concentration Ratio

The combined wet maize DDGS and MGM (50% DDGS + 50% MGM) were supplemented with varying doses of 0, 15, 45, 75, 105, 135, or 165 mg/kg of cellulase. Fermentation transpired under controlled settings at 37 °C with 55% humidity for a duration of 48 h. The study aimed to evaluate the effect of different enzyme–substrate ratios on the DMS and the levels of reducing sugars during the SSF of maize DDGS and MGM. All procedures followed the protocol described in Section 2.5. This experiment was repeated three times.

#### 2.5.2. Single-Factor Optimization of X1 Enzyme–Substrate Ratio

The mixed wet-based maize DDGS and MGM (50% DDGS + 50% MGM) were prepared, incorporating various concentrations of the X1 enzyme (0, 15, 30, 45, 60, 75, and 90 mg/kg). Fermentation was conducted under controlled conditions at 37 °C with 55% humidity for 48 h. This study aimed to evaluate the effect of differing X1 enzyme–substrate ratios on the DMS and the concentration of reducing sugars during the SSF of wet-grade maize DDGS and MGM. All procedures followed the protocol described in Section 2.5. This experiment was repeated three times.

#### 2.5.3. Single-Factor Optimization of Fermentation Time

Wet maize DDGS and MGM (50% DDGS + 50% MGM) were amalgamated and augmented with 75 mg/kg of cellulase and 60 mg/kg of X1. Fermentation was performed under controlled conditions at 37 °C with 55% humidity for periods of 24, 48, 72, or 96 h. This study investigated the effects of varying fermentation durations on the DMS and the concentration of reducing sugars during the SSF of wet-grade maize DDGS and MGM. All procedures followed the protocol described in Section 2.5. This experiment was repeated three times.

#### 2.5.4. SSF of Wet Maize DDGS-MGM Response Surface Experiment

On the basis of a single-factor test, a three-level, three-factor response surface methodology was designed. The experimental parameters are delineated in Table 1. All procedures were executed in accordance with the methodology defined in Section 2.5.1 of the optimization of the cellulase enzyme–substrate ratio.

### 2.6. Metabolism Experiment

#### 2.6.1. Animals, Diet, and Manure Collection Procedures

Forty 22-day-old Arbor Acres male broilers (body weight, 980.00 ± 5.10 g), exhibiting comparable body weights, were randomly assigned to five groups, each including eight duplicates with one broiler per replicate. The control group was administered a basal diet, while the experimental groups were provided with either an unfermented DDGS-MGM diet or a fermented DDGS-MGM diet, each replacing 30% of the basal diet. The grouping and treatment of experimental animals are shown in Table 2. The determination of dietary ME was strictly conducted in accordance with the technical specifications for ME determination of feed ingredients in white-feathered broilers issued by the Bureau of Animal Husbandry and Veterinary Medicine, Ministry of Agriculture and Rural Affairs of China (ME was estimated using the difference method) [29]. A three-day pre-feeding period was implemented to acclimate the broilers. Starting at 25 days of age, broilers had a 17 h feed withdrawal, after which they received the specified experimental diets. Total excreta collection was initiated simultaneously. Feeding continued for 55 h, after which feed intake was evaluated. The excreta collection was collected using the total collection method, with the collection period extended by an additional 17 h, resulting in a total period of 72 h. The terminal formula for white-feathered broilers is delineated in Table 3. Water was provided ad libitum via nipple-type drinkers, and feed was available freely. The nutritional composition of the basal diet was designed in accordance with the recommendations of the broiler feeding standard NY/T 33-2004 [30]. The ingredients and nutritional levels of the basal diet are presented in Table 3. The fermented DDGS-MGM was treated as a section of the 2.5 SSF of the wet maize DDGS-MGM response surface experiment.

#### 2.6.2. Quantification of Reducing Sugars Using the Dinitrosalicylic Acid Method

The sample was centrifuged after enzyme inactivation to extract the supernatant, and the concentration of reducing sugars in the supernatant was measured as described by Jain et al. [31].

#### 2.6.3. Sample Treatment and Chemical Analysis

The experimental diets were dried in an oven at 65 °C, and the broiler manure samples were placed in a freeze-dryer for freeze-drying. All samples were crushed for use. The moisture in diet and excreta was determined according to the Chinese national standard method of GB/T 6435-2014 [32], and the crude protein was determined according to GB/T 6432-2018 [33] and ISO 9831:1998 [34], respectively. The gross energy (GE) of diets and excreta was measured according to the method of ISO 9831:1998 using a Parr 6400 automatic adiabatic calorimeter (Parr Instrument Company, Moline, IL, USA). Nitrogen (N) content in diet and excreta samples was determined according to AOAC 1990 [35] using a Kjeldahl nitrogen analyzer (model KDY-9820, Shandong Haineng Scientific Instruments Co., Ltd., Dezhou, China), with crude protein (CP) calculated as N × 6.25.

#### 2.6.4. Determination of DMS and Apparent ME

The dry matter content was measured according to the national standard method AOAC 2001-12 [36].

DMS % = (dry matter content before sample pretreatment − dry matter content after sample pretreatment)/dry matter content before sample pretreatment

The determination method of metabolic energy was carried out in strict accordance with the standard [29], and the ME of feed ingredients was calculated according to the formula of Kong and Adeola [37].

Apparent metabolizable energy (AME) (MJ/kg) = [feed intake (kg) × feed energy (MJ/kg) − fecal volume (kg) × excreta energy (MJ/kg)]/feed intake (kg)

Metabolic energy calculation of the tested feed ingredient = [experimental diet metabolic energy − (100 − proportion of the tested feed ingredient)/100] × basal diet metabolic energy/proportion of the tested feed ingredient (%)

RN (Retained nitrogen, kg/kg feed intake) = Nitrogen intake − Nitrogen in excreta = Feed intake (kg) × Feed N content (%) − Output in excreta (kg) × N content in excreta (%)

AMEn (MJ × kg^−1^ DM) =AME (MJ × kg^−1^ DM) – RN × 34.39 (MJ/kg DM)

#### 2.6.5. Analysis Using Fourier Transform Infrared Spectroscopy

The structure of cellulose samples was characterized using Fourier transform infrared spectroscopy (FTIR) with a Nicolet Is50 FI-IR spectrometer from Thermo Fisher Scientific (Waltham, MA, USA). The sample was prepared using KBr mixed dilution tableting. The spectral resolution is 0.4 cm^−1^, and the scanning wavelength range extends from 400 to 4000 cm^−1^. The FTIR spectra of maize fiber displayed large peaks at 3371 cm^−1^ and 2923 cm^−1^, corresponding to the O-H stretching vibration of hemicellulose (methyl and methylene groups) and the C-H stretching vibration, respectively. An absorption peak at 1035 cm^−1^ was attributed to the C-O-C stretching of the glycosidic bond in xylan. The peak at 1727 cm^−1^ corresponded to the ester bond of ferulic acid, while the stretching absorptions at 1636 cm^−1^ (asymmetric COO^−^) and 1371 cm^−1^ (symmetric COO^−^) revealed the existence of uronic acid in the hemicellulose isolated from maize fiber [38]. Since DDGS and MGM were distinct by-products, inherent differences existed in their detailed fiber composition compared to raw corn fiber. Furthermore, factors such as sample preparation protocols, instrument calibration, and data processing methods may also contribute to variations in FTIR absorption peak positions.

#### 2.6.6. Determination of Monosaccharides

Nine milliliters of supernatant from enzymatic hydrolysis were transferred to a centrifuge tube, to which one milliliter of 10% sulfuric acid solution was added, mixed thoroughly, and allowed to stand for 10 min. The 2 mL mixture underwent centrifugation, and 1.5 mL of the supernatant was collected and neutralized with 4 M NaOH. We withdrew 100 μL of the neutralized sample, incorporated 50 μL of 1 M NaOH, added 250 μL of 1-phenyl-3-methyl-5-pyrazolone (PMP) derivative solution, and incubated this in a water bath at 70 °C for 60 min. Following cooling, it was neutralized with 50 μL of hydrochloric acid and subsequently dried. Subsequently, 500 μL of distilled water was incorporated for redissolution, followed by the addition of 700 μL of chloroform. The sample was vortexed by a vortex instrument for 1 min and then placed in a centrifuge at 6790× *g* for 3 min, and the procedure was repeated thrice. And then, 200 μL of the upper aqueous phase was extracted, and 600 μL of methanol was incorporated. The sample was vortexed for 1 min and centrifuged at 6790× *g* for 3 min, and the supernatant was transferred to the sample bottle and subjected to liquid chromatographic analysis using the Agilent 1260 Infinity II equipment (Munich, Germany). The mobile phases consisted of (A) a 1/1000 aqueous solution of formic acid, and (B) a 1/1000 solution of formic acid in acetonitrile. The flow rate was 0.700 mL/min, the chromatographic column used in this study was Eclipse XDB-C18 chromatographic column (i.d., 3.5 μm, Agilent Technologies Inc., Santa Clara, CA, USA), and the specific mobile phase protocol is detailed in Table 4. In this study, monosaccharides were detected using PMP derivatization, introducing two chromophores per sugar molecule. They were separated on a superficially porous C18 reversed-phase column using conventional liquid chromatography equipment, achieving a short analysis time (under 30 min) and high sensitivity (limit of quantification: 1 μg/mL), according to the standard of GB/T 40980-2021 [39].

Glucose, arabinose, mannose, and xylose standards (Shanghai Yuanye Bio-Technology Co., Ltd., Shanghai, China) were weighed, and the mixed standard solution of each monosaccharide standard was prepared and diluted to different concentrations. Four gradient standard solutions were prepared for each monosaccharide, which were 0.01, 0.1, 0.25, 0.5, 0.75, and 1 mg/mL, respectively. According to the retention time of each monosaccharide standard, the components were determined. Then the standard curve of each monosaccharide was drawn according to the peak area of each component. A linear standard curve equation was established based on the peak area and monosaccharide concentration in the liquid phase results. And the monosaccharide content in the supernatant of the test sample was calculated according to the peak area in the liquid phase results and the standard curve equation.

#### 2.6.7. Statistical Analysis

Statistical analyses were conducted utilizing SPSS 20.0 (IBM SPSS, Armonk, NY, USA). And data (including enzyme optimization trials along with animal trials for poultry ME determination) were expressed as mean ± standard error of the mean (SEM), with each experiment independently duplicated three times. The Shapiro–Wilk test confirmed the normality of all data (*p* > 0.05). Levene’s test was employed to evaluate the homogeneity of variances. For data that satisfied the assumption of homogeneity of variances (*p* > 0.05), one-way analysis of variance (ANOVA) was employed, followed by the least significant difference or Duncan post hoc test. In cases where the assumption of homogeneity of variance was not met (*p* < 0.05), Welch’s ANOVA was utilized, followed by Tamhane’s T2 post hoc test. Statistical significance was established at *p* < 0.05 unless specified otherwise.

The response surface test of 3 factors and 3 levels was designed by Box–Behnken in Design-Expert V8.0.6 software (Stat-Ease, Inc., Minneapolis, MN, USA) to optimize the parameters of DDGS-MDM pretreatment of wet maize. The quadratic regression equation was fitted with the DMS R1 as the response value, and the ternary quadratic regression equation of the DMS R1 to each influencing factor A (cellulase enzyme bottom ratio), B (X1 enzyme bottom ratio), and C (fermentation time) was obtained.

## 3. Results

### 3.1. Optimization of Pre-Digestion Conditions for Insoluble Fiber in Maize DDGS and MGM, and Economic Benefit Analysis

#### 3.1.1. Optimization of Pre-Digestion Conditions for Insoluble Fiber in Maize DDGS

In the pretreatment phase, four enzymes (cellulase, laccase, feruloyl esterase, and pectinase) were added simultaneously, and their enzyme-to-substrate ratios were sequentially optimized. During the pretreatment of maize DDGS or MGM insoluble fiber, the purpose of measuring DMS and reducing sugars served a dual purpose. Firstly, various enzymes were employed to deconstruct the insoluble fiber, disrupting the interwoven matrix of cellulose, hemicellulose, lignin, and other components. Secondly, enzymes such as cellulases were utilized to hydrolyze the fiber, both within and from its structure, into small molecules like monosaccharides or oligosaccharides, thereby providing energy for the animal organism. However, when the DMS and the optimal addition amount of reducing sugar were inconsistent, the DMS was given priority.

**Figure 1:**
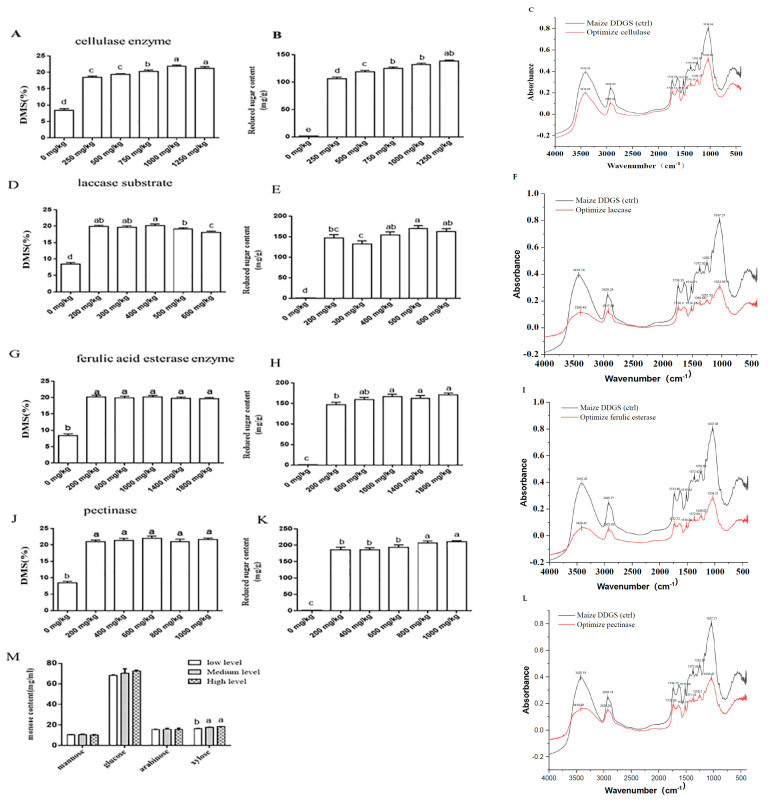
Optimization of the four enzymes’ enzyme-to-substrate ratios on the DMS, reducing sugar concentration, and structural characteristics of insoluble fiber in maize DDGS. (**A**,**D**,**G**,**J**) Effects of different enzyme–substrate ratios’ cellulase, laccase, ferulic acid esterase, or pectinase treatment of maize DDGS insoluble fiber on the DMS. (**B**,**E**,**H**,**K**) Effects of cellulase, laccase, ferulic acid esterase, or pectinase treatment on reducing sugar content of maize DDGS insoluble fiber with different enzyme to substrate ratios. (**C**,**F**,**I**,**L**) FT-IR spectrum of maize DDGS insoluble fiber after cellulase, laccase, ferulic acid esterase, or pectinase treatment. (**M**) The content of monosaccharides in the supernatant of maize DDGS insoluble fiber after enzymatic hydrolysis was optimized by the optimal ratio of four enzymes. Low level, 1000 mg/kg of cellulase, 200 mg/kg of laccase, 200 mg/kg of feruloyl esterase, and 400 mg/kg of pectinase; medium level, 1000 mg/kg of cellulase, 200 mg/kg of laccase, 200 mg/kg of feruloyl esterase, and 600 mg/kg of pectinase; high level, 1000 mg/kg of cellulase, 200 mg/kg of laccase, 200 mg/kg of feruloyl esterase, and 800 mg/kg of pectinase. Diverse superscripts ^(a–d)^ indicate significant group differences (*p* < 0.05). DMS, dry matter solubility.

Compared to the control group (inactivated enzymes), optimization of these ratios significantly increased DMS and reducing sugar yield (*p* < 0.05). As shown in Figure 1A,B, with the gradual increase in the cellulase-to-substrate ratio, DMS and reducing sugar yield from insoluble fibers in maize DDGS showed an initial continuous upward trend, which stabilized when the cellulase-to-substrate ratio reached 1000 mg/kg. Compared to other ratios (0, 250, 500, or 750 mg/kg), a cellulase-to-substrate ratio of 1000 mg/kg significantly improved DMS (*p* < 0.05) and increased reducing sugar production in the enzymatic hydrolysate supernatant (*p* < 0.05), while these two indicators were not changed compared with 1250 mg/kg of cellulase (*p* > 0.05). Comprehensively, the optimal cellulase-to-substrate ratio was determined as 1000 mg/kg. FTIR analysis confirmed the effective degradation of fibrous structures in maize DDGS by cellulase treatment. A significant decrease in absorbance was observed in the polysaccharide fingerprint region (1000–1100 cm^−1^), corresponding to C–O and C–O–C stretching vibrations in cellulose and hemicellulose, suggesting the hydrolysis of glycosidic bonds. A reduction in the ester bond region (~1735 cm^−1^) suggested partial cleavage of ferulic acid cross-links. The lignin-specific band at ~1510 cm^−1^ remained largely unchanged, indicating limited direct impact on the lignin structure. These results demonstrate that cellulase primarily targets polysaccharide components in DDGS, effectively disrupting the cell wall matrix (Figure 1C).

As the laccase-to-substrate ratio increased, the DMS of insoluble fibers in maize DDGS exhibited a continuous decline at 200 mg/kg, while the reducing sugar yield initially increased and then plateaued (Figure 1D,E). Compared to other groups (0, 300, 400, 500, or 600 mg/kg), a laccase-to-substrate ratio of 200 mg/kg achieved the highest DMS and reducing sugar yield (*p* < 0.05). Comprehensively, the optimal laccase-to-substrate ratio was determined as 200 mg/kg. Furthermore, FTIR analysis demonstrated that laccase treatment specifically modified the lignin structure in maize DDGS. A significant decrease in absorbance was observed at 1510 cm^−1^, corresponding to aromatic skeletal vibrations in lignin, indicating oxidative depolymerization. The reduction at 1240–1260 cm^−1^ (C–O stretching in guaiacyl units) further supported lignin degradation. In contrast, the polysaccharide region (1000–1100 cm^−1^) and ester bond region (~1735 cm^−1^) showed minimal changes, suggesting laccase’s selective action on lignin components. These results indicate that laccase effectively disrupts the lignin network without significantly affecting carbohydrate structures (Figure 1F).

With increasing feruloyl esterase-to-substrate ratios, the DMS of insoluble fibers in maize DDGS remained unchanged at 200 mg/kg of ferulic acid esterase, while the reducing sugar content initially increased and then stabilized at 159.58 mg/g (Figure 1G,H). A feruloyl esterase-to-substrate ratio of 600 mg/kg yielded the maximum reducing sugar content, compared with other esterase-to-substrate ratios (0, 200, 1000, 1400, or 1800 mg/kg) (*p* < 0.05). However, no significant differences were observed compared to the 200 mg/kg ratio (*p* > 0.05). Comprehensively, the optimal feruloyl esterase-to-substrate ratio was determined as 200 mg/kg. FTIR analysis demonstrated the specific efficacy of ferulic acid esterase treatment on maize DDGS. The most significant change was observed at 1735 cm^−1^, which corresponds to the C=O stretching vibration of ester bonds, particularly the ferulic acid cross-links between lignin and hemicellulose. A substantial decrease in absorbance at this wavelength suggested the targeted hydrolysis of these key cross-linking bonds by the ferulic acid esterase. Minor changes in the aromatic lignin region (around 1510 cm^−1^) and the polysaccharide fingerprint region (1000–1100 cm^−1^) indicate that the enzyme’s action was predominantly specific to ester bond cleavage rather than extensive degradation of the lignin aromatic structure or carbohydrate backbone. These results verify that ferulic acid esterase treatment effectively disrupts the lignin–carbohydrate complex network by releasing ferulic acid bridges, thereby increasing cell wall accessibility (Figure 1I).

As the pectinase-to-substrate ratio increased, the DMS of insoluble fibers in maize DDGS remained unchanged (*p* > 0.05) up to an enzyme-to-substrate ratio of 200 mg/kg, while the reducing sugar yield progressively increased up to a ratio of 800 mg/kg (Figure 1J,K). The administration of pectinase at varying enzyme–substrate ratios (0, 200, 400, 600, 800, and 1000 mg/kg) markedly increased the reducing sugar concentration in the supernatant of insoluble fiber from maize DDGS (Figure 1J,K). The reducing sugar content exhibited a consistent increase with elevated pectinase-to-substrate ratios, with the best ratio determined to be 800 mg/kg. FTIR analysis confirmed the specific degradation of pectic substances in maize DDGS following pectinase treatment. The most pronounced change was observed in the ester bond region (~1735 cm^−1^), which exhibited a significant decrease in absorbance, indicating the hydrolysis of methyl-esterified groups in pectin. Additionally, a noticeable reduction in the 1040–1100 cm^−1^ range (C–O and C–O–C stretching of polygalacturonic acid chains) further demonstrated the cleavage of the pectin backbone. In contrast, bands associated with lignin (~1510 cm^−1^) and typical cellulose/hemicellulose regions remained largely unchanged, highlighting the selective action of pectinase. These results indicate that pectinase effectively targets and degrades pectic polysaccharides, thereby promoting the disruption of the maize DDGS cell wall matrix and improving nutrient accessibility (Figure 1L). These results suggested that pectinase efficiently facilitated the breakdown of the insoluble fiber matrix in maize DDGS.

In summary, based on DMS and reducing sugar yield, the optimal enzyme combination for hydrolyzing insoluble fibers in maize DDGS was determined as 1000 mg/kg of cellulase, 200 mg/kg of laccase, 200 mg/kg of feruloyl esterase, and 800 mg/kg of pectinase. Following sequential optimization of enzyme-to-substrate ratios, four monosaccharides (including mannose, glucose, arabinose, and xylose) were detected in the enzymatic hydrolysate supernatant of maize DDGS insoluble fibers. Notably, xylose content increased progressively with escalating pectinase-to-substrate ratios (low, medium, and high levels were 400, 600, and 800 mg/kg, respectively) (Figure 1M).

#### 3.1.2. Optimization of Pre-Digestion Conditions for Insoluble Fiber in MGM

**Figure 2:**
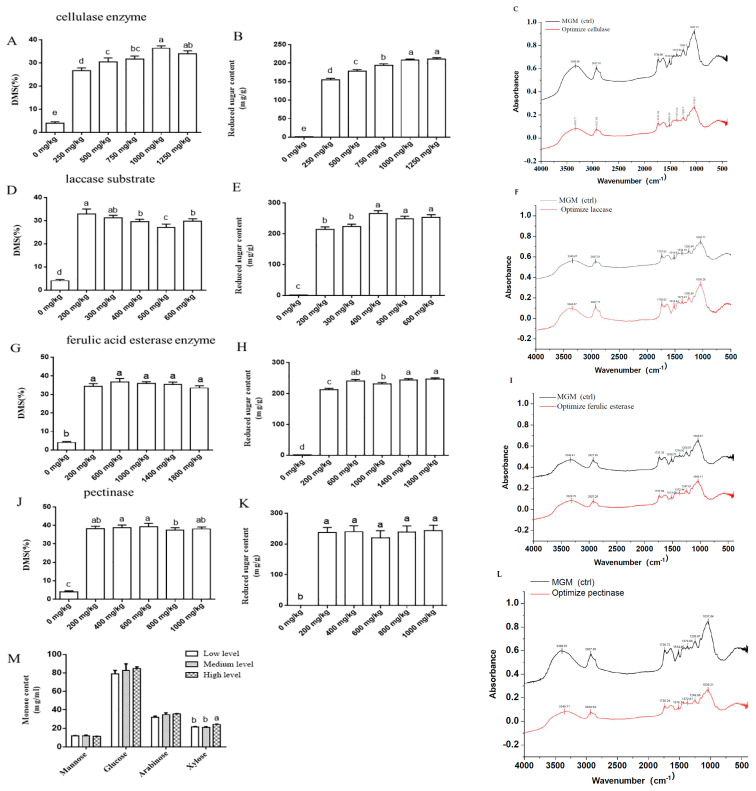
Optimization of the four enzymes’ enzyme-to-substrate ratios on the DMS, reducing sugar concentration, and structural characteristics of insoluble fiber in MGM. (**A**,**D**,**G**,**J**) The effects of different enzyme–substrate ratios’ cellulase, laccase, ferulic acid esterase or pectinase treatment of MGM insoluble fiber on the DMS. (**B**,**E**,**H**,**K**) Effect of cellulase, laccase, ferulic acid esterase, or pectinase treatment on reducing sugar content of MGM insoluble fiber with different enzyme-to-substrate ratios. (**C**,**F**,**I**,**L**) FT-IR spectrum of MGM insoluble fiber after cellulase, laccase, ferulic acid esterase, or pectinase treatment. (**M**) The content of monosaccharides in the supernatant of MGM insoluble fiber after enzymatic hydrolysis was optimized by the optimal ratio of four enzymes. Low level, 1000 mg/kg of cellulase, 200 mg/kg of laccase, 600 mg/kg of feruloyl esterase, and 200 mg/kg of pectinase; medium level, 1000 mg/kg of cellulase, 200 mg/kg of laccase, 200 mg/kg of feruloyl esterase, and 400 mg/kg of pectinase; high level, 1000 mg/kg of cellulase, 200 mg/kg of laccase, 200 mg/kg of feruloyl esterase, and 600 mg/kg of pectinase. Diverse superscripts ^(a–e)^ indicate significant group differences (*p* < 0.05). DMS, dry matter solubility.

In this study, four enzymes (cellulase, laccase, feruloyl esterase, and pectinase) were simultaneously added during pretreatment, and their enzyme-to-substrate ratios were sequentially optimized. As shown in Figure 2A,B, with increasing cellulase-to-substrate ratios, both DMS and reducing sugar yield from insoluble fibers in MGM exhibited a trend of continuous increase, which eventually stabilized at a ratio of 1000 mg/kg. Compared to ratios of 0, 250, 500, and 750 mg/kg, a cellulase-to-substrate ratio of 1000 mg/kg significantly improved DMS in MGM and elevated reducing sugar content in the supernatant (*p* < 0.05), while these two indicators were not changed compared with 1250 mg/kg of cellulase (*p* > 0.05) (Figure 2A,B). Therefore, the optimal cellulase-to-substrate ratio was determined as 1000 mg/kg. FTIR analysis demonstrated the effective degradation of fibrous structures in MGM after cellulase treatment. A significant decrease in absorbance was observed in the polysaccharide fingerprint region (1000–1100 cm^−1^), corresponding to C–O and C–O–C stretching vibrations in cellulose and hemicellulose, suggesting the hydrolysis of glycosidic bonds. A reduction in the ester bond region (~1735 cm^−1^) suggested partial cleavage of ferulic acid cross-links between lignin and carbohydrates. In contrast, the lignin-specific band at ~1510 cm^−1^ remained largely unchanged, indicating limited direct impact on the aromatic lignin structure. These results highlight that cellulase primarily targets polysaccharide components in MGM, effectively disrupting the cell wall matrix and enhancing nutrient availability (Figure 2C).

An increase in the laccase-to-substrate ratio resulted in divergent responses. The DMS of MGM insoluble fibers first increased at a ratio of 200 mg/kg or 300 mg/kg and then decreased at a ratio of 400 mg/kg, whereas the reducing sugar yield initially rose and subsequently stabilized at 224.12 mg/g (a ratio of 400 mg/kg) (Figure 2D,E). Compared to groups with ratios of 0, 400, 500, and 600 mg/kg, laccase at 200 or 300 mg/kg significantly enhanced DMS in MGM insoluble fibers (*p* < 0.05). Similarly, laccase at 400, 500, or 600 mg/kg significantly elevated the reducing sugar content in the enzymatic hydrolysate supernatant compared to ratios of 200 or 300 mg/kg (*p* < 0.05). Based on a comprehensive evaluation (prioritizing fiber structural deconstruction), the optimal laccase-to-substrate ratio was determined as 200 mg/kg. FTIR analysis confirmed the selective modification of the lignin structure in MGM following laccase treatment. A pronounced decrease in absorbance was observed at 1510 cm^−1^, corresponding to aromatic skeletal vibrations of lignin, indicating oxidative depolymerization of lignin subunits. Additional reduction in the 1240–1260 cm^−1^ range (C–O stretching of guaiacyl/syringyl units) further supported lignin degradation. In contrast, the polysaccharide-specific region (1000–1100 cm^−1^) remained largely unchanged, demonstrating the enzyme’s specificity toward lignin. These results indicate that laccase effectively disrupts the lignin network in MGM, potentially enhancing the accessibility of encapsulated nutrients (Figure 2F).

Feruloyl esterase-to-substrate ratios (0, 200, 600, 1000, 1400, and 1800 mg/kg) improved the reducing sugar content in the enzymatic hydrolysate supernatant of MGM insoluble fibers (Figure 2G,H). With increasing feruloyl esterase-to-substrate ratios, reducing sugar content in the hydrolysate exhibited a progressive upward trend, while DMS remained unchanged at a ratio of 200 mg/kg (*p* > 0.05). The 600 mg/kg ratio significantly elevated the reducing sugar content compared to the 200 mg/kg ratio (*p* < 0.05), with no other significant differences detected among the remaining ratios (*p* > 0.05). Comprehensively, the optimal feruloyl esterase-to-substrate ratio was determined as 600 mg/kg. FTIR spectra revealed a selective decrease in absorbance at 1735 cm^−1^ after ferulic acid esterase treatment, indicating cleavage of ferulic acid ester bonds in MGM. No significant changes were observed in lignin-associated (1510 cm^−1^) or polysaccharide-specific (1000–1100 cm^−1^) regions, demonstrating the enzyme’s targeted activity on ester linkages without broader cell wall degradation (Figure 2I).

As the pectinase-to-substrate ratio increased (0 to 1000 mg/kg), the dry matter solubility DMS of MGM insoluble fibers transitioned from a stable phase to a gradual decline at a ratio of 200 mg/kg, whereas the reducing sugar content showed no significant variation (*p* > 0.05, Figure 2J,K). Comprehensively, the optimal pectinase-to-substrate ratio was determined as 200 mg/kg compared with pectinase-to-substrate ratios. FTIR analysis showed a clear decrease in absorbance at 1740 cm^−1^ after pectinase treatment, indicating hydrolysis of methyl-esterified pectin in MGM. The 1050–1100 cm^−1^ region (C-O-C stretching) also showed reduced intensity, suggesting cleavage of polygalacturonic acid chains. No significant changes were observed in lignin (1510 cm^−1^) or cellulose-specific regions, suggesting pectinase’s specific activity on pectin components (Figure 2L).

In summary, based on DMS and reducing sugar yield, the optimal enzyme combination for hydrolyzing MGM insoluble fibers was identified as 1000 mg/kg of cellulase, 200 mg/kg of laccase, 600 mg/kg of feruloyl esterase, and 200 mg/kg of pectinase. Liquid chromatography results demonstrated that the reducing sugars in the enzymatic hydrolysate supernatant of MGM insoluble fibers, following optimization of cellulase–laccase–feruloyl esterase–pectinase enzyme-to-substrate ratios, were primarily composed of mannose, glucose, arabinose, and xylose, with glucose being the predominant component. Compared to the low-dose (200 mg/kg) or medium-dose (400 mg/kg) group, high-dose (600 mg/kg) pectinase optimization significantly increased xylose content in the hydrolysate supernatant (Figure 2M) (*p* < 0.05).

#### 3.1.3. Optimization of Cellulase and X1 Synergistic Pre-Digestion Parameters for Insoluble Fibers in Maize DDGS and MGM

**Figure 3:**
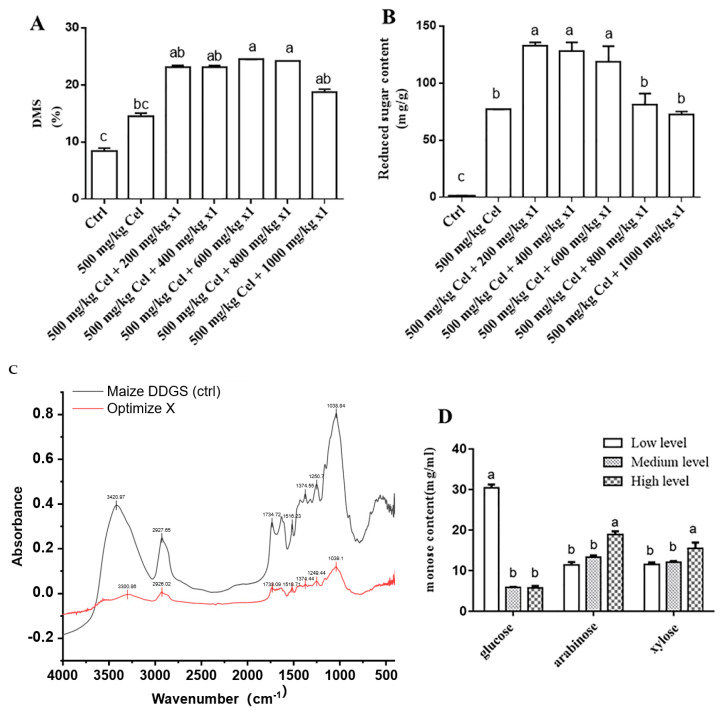
The changes in insoluble fiber DMS, reducing sugar, structure, and monosaccharide in maize DDGS were optimized by the X1 enzyme–substrate ratio. (**A**) Effects of different enzyme–substrate ratio X1 on DMS of maize DDGS insoluble fiber treated with X1. (**B**) Effect of different enzyme–substrate ratio X1 on reducing sugar content of maize DDGS insoluble fiber. (**C**) FT-IR spectra of insoluble fiber of maize DDGS treated by X1. (**D**) Effect of X1 enzyme combined with cellulase on the supernatant of maize DDGS insoluble fiber. Low level, 500 mg/kg of cellulase and 200 mg/kg of X1 enzyme; medium level, 500 mg/kg of cellulase and 400 mg/kg of X1 enzyme; high level, 500 mg/kg of cellulase and 600 mg/kg of X1 enzyme. Diverse superscripts ^(a–c)^ indicate significant group differences (*p* < 0.05). DMS, dry matter solubility.

Due to the partial cellulase activity inherent in the X1 enzyme, its combined application with 1000 mg/kg or 500 mg/kg of cellulase yielded equivalent effects at a fixed X1 dosage. Considering cost efficiency, 500 mg/kg of cellulase was selected for subsequent trials. Under a constant cellulase dosage (500 mg/kg), the addition of 200 mg/kg of X1 significantly increased DMS to 24.26% and elevated reducing sugar content to 132.58 mg/g in maize DDGS insoluble fibers compared to cellulase alone (*p* < 0.05) (Figure 3A,B). However, higher X1 dosages markedly reduced the reducing sugar content in the hydrolysate supernatant (*p* < 0.05). FTIR analysis demonstrated the multi-enzymatic activity of the X1 enzyme complex on maize DDGS. A broad reduction in absorbance was observed across key functional groups: a decrease at 1735 cm^−1^ indicated cleavage of ester bonds (e.g., ferulic acid cross-links), while a significant reduction in the 1000–1100 cm^−1^ region confirmed extensive degradation of cellulose and hemicellulose glycosidic bonds. Minor changes in the lignin aromatic region (1510 cm^−1^) suggested limited direct lignin modification. The simultaneous degradation of multiple structural components highlights the synergistic action of the X1 enzyme blend in effectively disrupting the fibrous matrix (Figure 3C). Synergistic hydrolysis with X1 and Cel released glucose, arabinose, and xylose in the hydrolysate supernatant. Notably, glucose content exhibited a decreasing trend with increasing X1 enzyme dosage (200, 400, 600 mg/kg) (Figure 3D), which may be attributed to trace amounts of *Aspergillus niger* spores present in the solid-state-fermented X1 enzyme preparation (Figure 3D).

**Figure 4:**
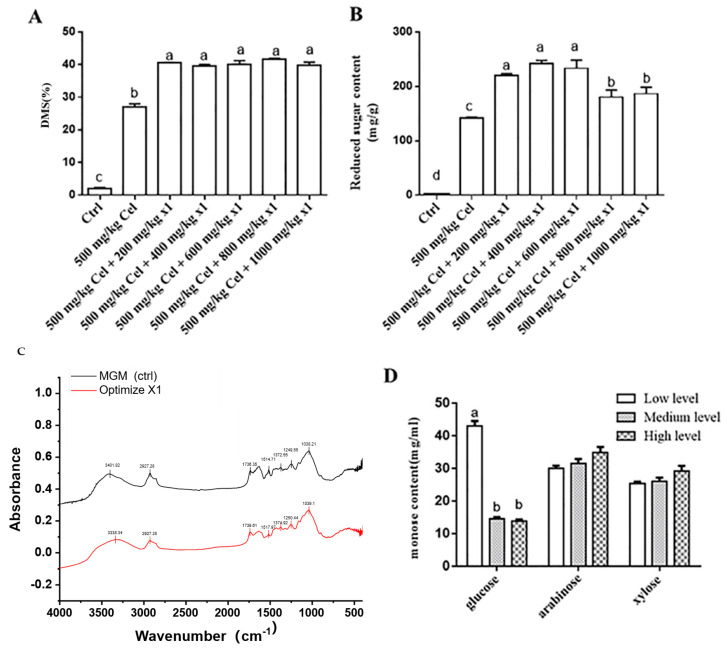
The changes in insoluble fiber DMS, reducing sugar, structure, and monosaccharide in MGM were optimized by the X1 enzyme–substrate ratio. (**A**) Effects of different enzyme–substrate ratios’ X1 enzyme on the DMS of insoluble fiber in MGM. (**B**) Effect of insoluble fiber of MGM treated with different enzyme–substrate ratio X1 on reducing sugar content. (**C**) FT-IR spectra of insoluble fiber of MGM treated by X1. (**D**) Effect of X1 enzyme combined with cellulase on the supernatant of MGM insoluble fiber. Effect of X1 treatment of insoluble fiber of MGM on its supernatant. Low level, 500 mg/kg of cellulase and 200 mg/kg of X1 enzyme; medium level, 500 mg/kg of cellulase and 400 mg/kg X1 of enzyme; high level, 500 mg/kg of cellulase and 600 mg/kg of X1 enzyme. Diverse superscripts ^(a–d)^ indicate significant group differences (*p* < 0.05). DMS, dry matter solubility.

Under a fixed cellulase dosage (500 mg/kg), the addition of 200 mg/kg of X1 enzyme achieved a DMS of 41.17% (*p* < 0.05) and a reducing sugar content of 241.25 mg/g in MGM insoluble fibers (*p* < 0.05), compared with other ratios of enzyme to substrate (Figure 4A,B). FTIR analysis demonstrated the multi-enzymatic activity of the X1 enzyme complex on MGM. A broad reduction in absorbance was observed across key functional groups: a significant decrease at 1735 cm^−^^1^ indicated cleavage of ester bonds (e.g., ferulic acid cross-links), while a pronounced reduction in the 1000–1100 cm^−1^ region confirmed extensive degradation of cellulose and hemicellulose glycosidic bonds. Minor changes in the lignin aromatic region (1510 cm^−1^) suggested limited direct lignin modification. The simultaneous degradation of multiple structural components highlights the synergistic action of the X1 enzyme blend in effectively disrupting the MGM fibrous matrix (Figure 4C). Synergistic hydrolysis with X1 and cellulase released glucose, arabinose, and xylose in the enzymatic hydrolysate supernatant. Notably, glucose content exhibited a declining trend with increasing X1 dosage (200, 400, 600 mg/kg), potentially attributable to trace *Aspergillus niger* spores presented in the solid-state fermented X1 enzyme (Figure 4D).

#### 3.1.4. Economic Benefit Analysis

The unit prices of cellulase, laccase, ferulic acid esterase, pectinase, and the X1 enzyme involved in this study were 31.86 RMB/kg, 20.00 RMB/kg, 75.22 RMB/kg, 30.97 RMB/kg, and 20.00 RMB/kg. The two sets of process parameters and costs for the pretreatment of maize DDGS insoluble fiber were 1000 mg/kg cellulase + 200 mg/kg laccase + 200 mg/kg feruloyl esterase + 800 mg/kg pectinase, with a process cost of 75.68 RMB/kg; 500 mg/kg cellulase + 200 mg/kg X1 enzyme with a process cost of 19.93 RMB/kg. For pretreatment of MGM insoluble fiber, the corresponding parameters and costs were 1000 mg/kg cellulase + 200 mg/kg laccase + 600 mg/kg feruloyl esterase + 200 mg/kg pectinase at 87.19 RMB/kg; 500 mg/kg cellulase + 200 mg/kg X1 enzyme at 19.93 RMB/kg. Economic analysis revealed that the optimal pretreatment parameters for insoluble fiber in both raw materials were achieved through synergistic treatment with cellulase and the X1 enzyme.

Based on our unpublished research on fermented feed in broiler trials, the developed fermented DDGS-MGM product could replace 5% of the maize–soybean meal blend (specifically 2.9% maize and 2.1% soybean meal) in compound diets. Considering 2024 Chinese commodity prices for maize, soybean meal, DDGS, and MGM, along with operational costs including labor and equipment depreciation, substituting 5% maize–soybean meal with fermented DDGS-MGM in one metric ton of compound feed yields a net cost reduction of 20.45 RMB (Table 5).

### 3.2. Experimental Design and Optimization of SSF Conditions of Maize DDGS-MGM

Optimization of SSF conditions for maize DDGS- MGM mixtures using a single-factor experimental design was performed. Based on the results obtained from the initial screening phase, SSF of maize DDGS and MGM was initiated. Initially, single-factor optimization was conducted to determine the optimal conditions for cellulase and X1 dosage, as well as enzymatic hydrolysis time, with the DMS serving as the primary evaluation criterion. Subsequently, a validation experiment was performed to confirm the optimized conditions. Based on the evaluation of reducing sugar content and DMS, the optimal enzyme–substrate ratios for wet-grade DDGS-MGM were determined to be 75 mg/kg for cellulase and 60 mg/kg for X1 (Figure 4A,B).

#### 3.2.1. Optimization of SSF Conditions of Maize DDGS-MGM by Cellulase and X1 Enzyme

##### Single-Factor Optimization of Cellulase and X1 Enzyme–Substrate Ratio

**Figure 5:**
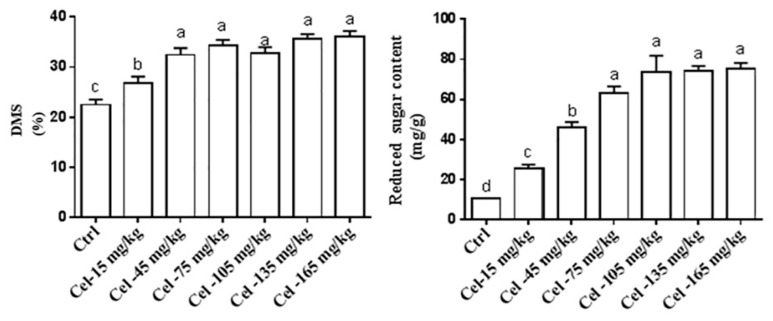
The effects of cellulase enzyme-to-substrate ratios (0, 15, 45, 75, 105, 135, or 165 mg/kg) on DMS and reducing sugar content in SSF of maize DDGS-MGM were optimized. DMS, dry matter solubility. Diverse superscripts ^(a–d)^ indicate significant group differences (*p* < 0.05).

Under identical SSF conditions, as the cellulase enzyme-to-substrate ratio increased, both the DMS of maize DDGS-MGM solid-state fermented material and the reducing sugar production in the supernatant exhibited a gradual increase followed by stabilization. Compared with the 15 mg/kg group, other cellulase enzyme-to-substrate ratio groups significantly enhanced DMS (*p* < 0.05). Relative to the 0, 15, and 45 mg/kg cellulase groups, the 75, 105, 135, and 165 mg/kg cellulase groups significantly increased reducing sugar production in the solid-state fermented material (*p* < 0.05). Comprehensive analysis of dry matter utilization efficiency and reducing sugar content indicated that the optimal enzymatic hydrolysis effect was achieved at a cellulase enzyme-to-substrate ratio of 75 mg/kg (Figure 5).

**Figure 6:**
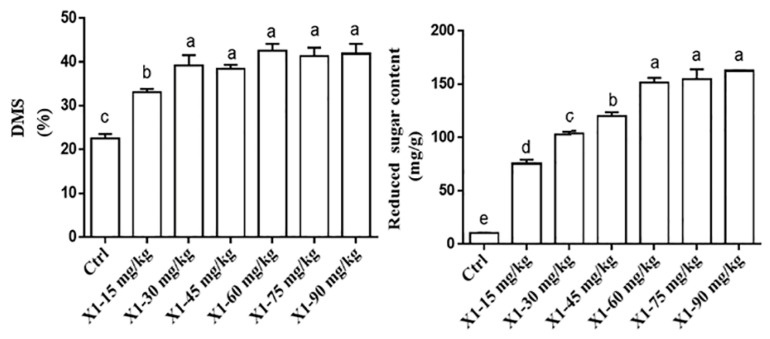
The effects of X1 enzyme–substrate ratios (0, 15, 30, 45, 60, 75, or 90 mg/kg) on DMS and reducing sugar content in maize DDGS-MGM by SSF were optimized. DMS, dry matter solubility. Diverse superscripts ^(a–e)^ indicate significant group differences (*p* < 0.05).

With standardized SSF settings, as the X1 enzyme-to-substrate ratio increased, both the DMS of maize DDGS-MGM solid-state fermented material and the reducing sugar production in the supernatant initially increased and then stabilized at 32.46% and 63.00 mg/g, respectively. Compared with the 0 and 15 mg/kg X1 enzyme groups, the 30, 45, 60, 75, and 90 mg/kg X1 groups significantly increased the DMS in the fermented material (*p* < 0.05). Relative to the 0, 15, 30, and 45 mg/kg X1 groups, the 60, 75, and 90 mg/kg X1 groups significantly enhanced the reducing sugar content in the enzymatic hydrolysate supernatant (*p* < 0.05). Comprehensive analyses of DMS and reducing sugar production demonstrated that the optimal enzymatic hydrolysis effect was achieved at an X1 enzyme-to-substrate ratio of 60 mg/kg (Figure 6).

##### Single-Factor Optimization of Fermentation Time

Under identical SSF conditions, the DMS of maize DDGS-MGM fermented substrates exhibited a gradual increasing trend with prolonged fermentation time, while the reducing sugar yield in the enzymatic hydrolysate supernatant demonstrated an initial increase followed by a decreasing trend. Compared with 24 h, 72 h, and 96 h fermentation durations, 48 h fermentation significantly enhanced the reducing sugar content (*p* < 0.05). Relative to 24 h, 48 h, and 72 h fermentation periods, 96 h fermentation achieved a significant improvement in DMS (*p* < 0.05). Comprehensive analysis of DMS and reducing sugar production identified optimal conditions at a cellulase dosage of 75 mg/kg, X1 enzyme–substrate ratio of 60 mg/kg, and fermentation duration of 48 h, yielding reducing sugar content of 200.26 mg/g and DMS reaching 48.77% (Table 6).

#### 3.2.2. Optimization of SSF Conditions by Response Surface Methodology

Based on the results of a single-factor experiment, the response surface experiment was designed by Design-Expert V8.0.6 Box–Behnken, with cellulase enzyme–substrate ratio (A), X1 enzyme–substrate ratio (B), and fermentation time (C) as experimental conditions. The model fitting analysis indicated a significant model with a *p*-value of 0.0076 (*p* < 0.05). The order of influence of the three factors on DMS was ranked as X1 enzyme–substrate ratio (B) > fermentation time (C) > cellulase enzyme–substrate ratio (A). The model exhibited a satisfactory coefficient of determination (R^2^ = 0.9048), and the lack-of-fit term was not significant, suggesting good fitting adequacy for analyzing and predicting DMS (Table 7). The reliability analysis demonstrated a low coefficient of variation (CV = 3.72%), supporting the experimental reliability (Table 8).

Based on experimental results, a quadratic regression model was established using DMS (R) as the response variable. The second-order polynomial regression equation for R1 with respect to the variables A (cellulase enzyme–substrate ratio), B (X1 enzyme–substrate ratio), and C (fermentation time) was formulated as follows:

R = 15.61250 + 0.020042 × A + 0.270656 × B + 0.3975 × C + 0.000319 × AB + 0.000222 × AC − 0.000833 × BC − 0.000174 × A^2^ − 0.001204 × B^2^ − 0.001803 × C^2^

**Figure 7:**
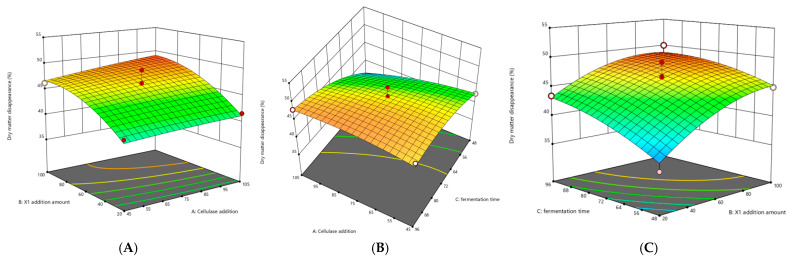
Response surface interaction of two factors. (**A**) The interaction between the cellulase–substrate ratio and X1 enzyme–substrate ratio. (**B**) The interaction between the cellulase–substrate ratio and fermentation time. (**C**) The interaction between the X1 enzyme–substrate ratio and fermentation time. The variation in color on the response surface illustrated the change in the predicted response. The gradient, from cool tones (blue, indicating lower values) to warm tones (red, indicating higher values), and the contour lines together facilitated the identification of the optimal region.

Response surface analysis visually demonstrated the magnitude of interaction effects on response variables: steeper curvature and denser contour lines indicate greater significance. The response surfaces for DMS (Figure 7) revealed significant pairwise interactions among the cellulase enzyme–substrate ratio, X1 enzyme–substrate ratio, and hydrolysis duration.

#### 3.2.3. Verification and Determination of Optimal Co-Fermentation Culture Conditions

The optimal fermentation conditions for DMS in solid-state fermented DDGS-MGM, as determined by Design-Expert V8.0.6 analysis, were identified as follows: cellulase enzyme–substrate ratio of 75 mg/kg, X1 enzyme–substrate ratio of 97 mg/kg, and fermentation duration of 82 h, achieving a DMS of 48.58%. Replicated validation experiments under these optimized conditions yielded a solubility of 49.10%, which closely aligned with theoretical predictions, supporting the reliability of the parameters obtained through response surface optimization.

### 3.3. Effects of SSF of Maize DDGS-MGM on Metabolic Energy of White Feather Broilers

Compared with the control group, the total feed intake value of the unfermented group (50%DDGS + 50%MGM) was lower (*p* < 0.05), and the values in other groups did not change (*p* > 0.05). From the index of GE, the GE value of fermented feed was lower than that of unfermented feed (*p* < 0.05). Compared to the non-fermented group (including unfermented group (50%DDGS + 50%MGM) or unfermented group (62.5%DDGS + 37.5%MGM), broilers fed fermented diets exhibited significantly higher AME, AMEn, AME/GE, and AMEn/GE, indicating that solid-state fermented maize DDGS-MGM enhanced energy metabolism efficiency in broilers (Table 9).

Relative to the non-fermented diet (50% DDGS + 50% MGM), fermented diets (50% DDGS + 50% MGM) significantly increased AME and AMEn (*p* < 0.05), with a 2.74 MJ/kg and 2.73 MJ/kg improvement (39.60% and 40.81% increase) observed in fermented diets (Table 9). Similarly, fermented diets (62.5% DDGS + 37.5% MGM) elevated AME and AMEn by 1.4 MJ/kg and 1.42 MJ/kg (16.02% and 16.68% increase) compared to non-fermented counterparts (62.5% DDGS + 37.5% MGM) (Table 10). These results suggested that higher DDGS dry matter content in substrates adversely affected the ME enhancement of solid-state fermented products in broilers.

## 4. Discussion

In this study, in situ pre-digestion process parameters were established for wet-based maize DDGS to improve its metabolic energy. Using insoluble fibers in DDGS as substrates, optimal parameters were identified as 500 mg/kg of cellulase synergized with 200 mg/kg of X1 enzyme through enzyme combination optimization and economic evaluation. Additionally, SSF of wet-base DDGS and MGM supplemented with cellulase, X1 enzyme, and xylose-utilizing *lactic acid bacteria* improved AME and AMEn in broiler diets (39.60% and 40.81%increase).

High-fiber feeds exhibited lower DMS, indicating a recalcitrant fiber structure (e.g., high lignification) that resisted efficient microbial breakdown. This resulted in lower nutritional value, necessitating greater feed intake by animals to meet nutritional requirements. A high DMS signified that the feed could provide more fermentable energy to meet the maintenance and growth needs of livestock and poultry. Given the structural complexity of maize fiber, utilizing multi-enzyme complexes for pretreatment represented the primary approach to degrade its fiber structure. Using insoluble fiber from maize DDGS and MGM as substrates, the synergistic action of cellulase, laccase, feruloyl esterase, and pectinase under the optimized parameters of this trial significantly increased both DMS and reducing sugar yield. The notable improvements could be explained by the strategic selection of enzymes tailored to the substrate. The efficacy of the selected enzymes—particularly the cellulase complex used in this study—was attributed to the match between their active sites and the distinctive chemical structure of maize fiber [28,40]. Efficient degradation of anti-nutritional factors in DDGS and MGM was achieved through synergistic interactions among specific enzymes. Cellulase targeted both crystalline and amorphous regions of cellulose, hydrolyzing β-1,4-glycosidic bonds to disrupt physical barriers and enhance substrate accessibility, leading to a marked decrease in DMS [41]. Laccase, utilizing its copper ion active center, catalyzed the oxidation of phenolic units in lignin under aerobic conditions, generating radical intermediates that initiated the depolymerization of macromolecular lignin and promoted the release of nutrients [42]. This enzyme played a notable role in maize fiber degradation, with reported solubility increases of 26% and 21% for maize stover and wheat straw, respectively [43]. Feruloyl esterase specifically hydrolyzed ester bonds linking hemicellulose to phenolic acids (e.g., feruloyl-arabinoxylan esters) and those connecting lignin to carbohydrates (e.g., feruloyl-lignin esters), thereby disrupting the cell wall network and releasing free ferulic acid [44,45]. A synergistic effect between feruloyl esterase and laccase was previously demonstrated [46,47]. Pectinase cleaves α-1,4-glycosidic bonds in the pectin backbone, generating short-chain galacturonic acids. It also contributes to reducing chyme viscosity by degrading pectin, which enhances nutrient diffusion and absorption [48]. Zhao et al. [49] demonstrated that pectinase acts synergistically with cellulase, hemicellulase, and laccase to improve reducing sugar yield from maize stover. The collective action of these enzymes significantly reduced anti-nutritional factors in DDGS and MGM. The lignocellulosic matrix was effectively dismantled, increasing the exposure of intracellular nutrients such as proteins and lipids, mitigating anti-nutritional effects, and ultimately improving the digestibility and energy availability of DDGS and MGM.

Furthermore, our study confirmed that the synergistic effect of cellulase and X1 enzyme in improving the DMS of maize DDGS and MGM was superior to that achieved by the combined pretreatment with cellulase, laccase, feruloyl esterase, and pectinase, with enhancements of 24.26% and 41.17%, respectively. The primary reason was that X1 enzyme, an SSF-derived enzymatic preparation (complex enzyme formulation), contained 5000 U/g of xylanase, 50 U/g of xylosidase, 100 U/g of endoglucanase, 150 U/g of glucosidase, and 300 U/g of pectinase. This enzyme demonstrated distinct advantages in degrading natural fibers such as maize DDGS and MGM. Glycoside hydrolases have been widely documented to play crucial roles in rice cell wall structure and function, cell expansion, and various other physiological processes. Han et al. [50] demonstrated that arabinoxylan in maize straw was highly substituted and resistant to microbial enzymatic degradation. Therefore, novel enzymes such as β-xylosidase—a key enzyme involved in plant cell wall modification—are required to hydrolyze hemicellulose in maize straw and improve its solubility. Consequently, future research should prioritize investigating the role and efficacy of β-xylosidase in degrading maize fiber materials such as DDGS and MGM.

To address the challenge of utilizing wet-base maize DDGS in feed, this study used a substrate comprising 50% wet-base maize DDGS (dry matter) and 50% dry-base MGM. Under 55% moisture content, SSF with cellulase synergized with enzyme X1 and *Lactobacillus strain* HX-31 achieved a DMS of 48.58% for the maize DDGS-MGM fermentation product. The selection of microbial strains and SSF parameters (aeration, moisture content, temperature, duration) critically influences the fermentation efficacy of feed ingredients. For microbial inoculation, this study employed a UV-mutated *Lactobacillus plantarum strain* HX-31, demonstrating enhanced lactic acid production through efficient xylose utilization. Both MGM substrates contain substantial xylan, which enzymatic pretreatment converts to xylose. This xylose is subsequently metabolized by *lactic acid bacteria* to xylulose via the pentose phosphate pathway, ultimately yielding lactic acid for broiler energy provision [51]. This could also explain the increased metabolic energy of DDGS-MGM in wet samples after fermentation. Anaerobic SSF was adopted, favoring the rapid accumulation of organic acids, probiotics, and microbial proteins. This is also why solid-state fermented feeds are gradually being widely promoted and applied. In addition, moisture content regulation during SSF serves as a critical parameter influencing microbial growth, metabolic efficiency, and product synthesis. Optimal moisture ranges vary with fermentation types and substrates: fungal-dominated processes (yeasts/molds) require 50–65% moisture for cellulase production, organic acid synthesis, or antibiotic biosynthesis; bacterial/actinobacterial fermentations operate at 40–55% for metabolite production; mixed-culture systems function optimally at 55–70% moisture for composting or biofuel applications [52]. Moisture content was maintained at 55% for mixed DDGS-MGM substrates, balancing microbial viability and nutrient diffusion. This level prevents cellular dehydration while enabling porous matrix formation for gas exchange and solute transport [53]. Suboptimal moisture (<50%) restricted substrate accessibility and promoted inhibitory metabolite accumulation, impairing microbial metabolism [54]. Fermentation temperature was optimized within the operational range of cellulase and X1 enzyme to maximize catalytic efficiency. Duration was calibrated against saccharification kinetics and microbial sugar consumption—insufficient time limits substrate utilization, while prolonged fermentation induces energy loss through microbial assimilation of oligosaccharides. Process optimization thus focuses on maximizing glucose and oligosaccharide yields for direct broiler energy utilization.

The degradation degree of dietary NDF was directly linked to feed energy availability. The NDF values of soluble maize bran and maize bran were 22.7% and 40.6%, respectively, and the energy values were 4.58 and 3.68 Mcal/kg, respectively, indicating that high DF will reduce the energy value [9]. It was established in prior research that NDF in animal feces is negatively correlated with ME in the diet (r = −0.74; *p* < 0.001) [55]. A study confirmed that using dry-based maize DDGS and wheat bran (80:20 ratio) as substrate, aerobic fermentation with *Bacillus subtilis* CW4 for 36 h (Stage 1) followed by anaerobic fermentation with Lactobacillus plantarum for 48 h (Stage 2) reduced cellulose, lignin, NDF, and ADF contents by 37.71%, 37.85%, 32.70%, and 52.27%, respectively [56]. And using wet-based maize DDGS with wheat bran (1:2, m/m; material-to-water ratio 1:3.5), fermented with *Trichoderma reesei* and *Candida utilis* (inoculation ratio 2:1) for 8 days, achieved 30.10% cellulose degradation, 13.10% hemicellulose degradation, and increased apparent dry matter digestibility to 62.8% [57]. This study demonstrated that enzyme–microbe synergy (cellulase, X1 enzyme, Lactobacillus HX-31) during SSF of wet-based maize DDGS with MGM (50%:50% dry basis) reduced NDF and increased ME. These collective findings indicated that enzyme/microbe species and fermentation protocols directly influence fermentation efficacy and consequently impact the energy value of fermented products.

The ME of feed was regulated by a combination of raw material properties, animal physiological status, and processing techniques [58]. In terms of feed ingredients, higher crude fiber content was generally associated with lower ME values [59], primarily due to the low utilization efficiency of cellulose and hemicellulose in monogastric animals. Regarding animal physiological characteristics, monogastric species such as poultry lacked the efficient microbial fiber degradation system present in ruminants, resulting in significantly limited fiber utilization capacity [60]. Processing technologies, particularly the application of enzyme preparations, markedly improved ME levels. For instance, in diets for 1~21-day-old Ross 308 broilers, the addition of 200 g/t or 400 g/t of a complex enzyme ASC (including phytase, pectinase, protease, β-glucanase, cellulase, amylase, and endo-1,4-β-xylanase) significantly increased AME, compensating for the 0.37 MJ/kg reduction in ME observed in the negative control group [61]. Another study also indicated that supplementing a maize–soybean meal diet with a multi-enzyme complex (containing xylanase, β-glucanase, cellulase, protease, pectinase, debranching enzymes, among others) led to a slight decrease in apparent ME during the initial phase but significantly improved the AMEn retention rate during the grower phase (26–35 days) and over the entire production cycle (0–35 days) [62]. Further studies supported the positive effects of enzymatic treatment on regulating ME. Wu et al. reported that although the addition of alkaline protease to a DDGS-based diet did not result in a statistically significant increase in AME (3784 kcal/kg vs. 3641 kcal/kg) and AMEn (3516 kcal/kg vs. 3393 kcal/kg), an upward trend was observed [63]. Romero et al. found that supplementing with an enzyme complex containing xylanase and amylase significantly increased AMEn by 2.22% (12.89 MJ/kg vs. 12.61 MJ/kg) [64]. In the present study, using a 50:50 mixture of DDGS and MGM subjected to SSF with enzymatic pretreatment, AME and AMEn significantly increased by 39.60% and 40.80%, respectively. This indicated that the combined use of wet fermentation technology and specific enzyme formulations (such as cellulase and X1 enzyme) played a key role in improving energy release from the fibrous matrix. Therefore, the rational selection of enzyme combinations and pretreatment methods effectively disrupted fiber structures and reduced anti-nutritional factors, thereby significantly enhancing the energy utilization efficiency of feed.

Fermentation of wet-base maize DDGS and MGM effectively enhanced both nutritional value and economic value, as evidenced by improvements in fiber degradation, ME, and economic assessments. Enzymatic pretreatment disrupted fiber structures and broke down non-starch polysaccharides, leading to a significant increase in substrate solubility and higher reducing sugar yield. FTIR analysis, together with measurements of DMS and reducing sugar content, collectively confirmed the effective degradation of cellulose and hemicellulose. Animal trials demonstrated significant increases in both AME and AMEn, indicating that fiber degradation products—such as oligosaccharides and organic acids—positively contributed to energy utilization [65,66]. Numerous studies reported that solid-state fermented feed could improve gut health in broilers [67,68]. In the later phase of this study, animal trials were conducted to evaluate the effects of replacing 5% of the maize–soybean meal diet with solid-state fermented feed—produced using parameters optimized in this study—on the intestinal tract. Future research should focus on systematically linking nutrient utilization to animal physiological responses—such as gut microbiota composition and metabolic profiles—to clarify the mechanism underlying “treatment–structural change–nutrient release–host response”. The integration of multi-omics technologies could provide further mechanistic insights to support process optimization and practical application. From an economic perspective, the utilization of 5% fermented DDGS-MGM in broiler diets to replace the conventional maize–soybean meal diet reduced the feed cost by 20.45 RMB per metric ton. It was suggested that further modification of the X1 enzyme or exploration of novel multifunctional enzymes for fermenting DDGS-MGM could lead to additional feed cost savings in the future.

Optimization at laboratory scale was essential for advancing SSF, yet scaling to industrial levels introduced major challenges. Substrate bed thickening impeded oxygen transfer and carbon dioxide dissipation, often resulting in anaerobic zones. Poor heat dissipation led to temperature non-uniformity and localized overheating, inhibiting microbial and enzymatic activity [69]. Online sensor deployment in large-scale reactors remained difficult, hindering real-time monitoring and dynamic control of critical parameters such as temperature, pH, and moisture [70,71]. Although SSF systems exhibited inherent contamination resistance, controlling microbial pollution under non-sterile conditions required stringent measures due to increased sterilization costs and operational complexity. Furthermore, from a compliance perspective, it was necessary to establish comprehensive standards covering raw materials, process monitoring, and final product testing, along with a fully traceable quality system to ensure every batch met regulatory requirements for safety, efficacy, and consistency. Addressing these challenges was believed to depend on smarter reactor designs and advanced control strategies. Following the efforts to address these scale-up challenges, we further evaluated the health benefits of the lab-scale solid-state fermented product on broiler intestinal function.

## 5. Conclusions

In summary, this study demonstrated that enzymatic pretreatment with specifically formulated cellulase and X1 enzyme blends significantly enhanced the degradability of maize DDGS and MGM insoluble fibers. The optimized SSF process for the DDGS-MGM substrate resulted in substantially improved DMS and a 39.60% increase in ME. Dietary incorporation of fermented DDGS-MGM at a 5% substitution level achieved a feed cost reduction of 20.45 RMB per metric ton, indicating potential economic benefits. These findings provide valuable insights into the efficient utilization of cereal processing by-products, suggesting promising strategies for enhancing feed value. However, further research is warranted to validate these outcomes under commercial production conditions and to investigate comprehensive nutrient utilization beyond energy evaluation.

## Figures and Tables

**Table 1:** Factors and levels of experimental design.

Factor	A Cellulase Enzyme–Substrate Ratio (mg/kg)	B X1 Enzyme Base Ratio (mg/kg)	C Fermentation Time (h)
Level-1	45	20	48
Level 0	75	60	72
Level 1	105	100	96

**Table 2:** Experimental animal grouping and dietary treatments.

Grouping	Treatment
1	Control group
2	Unfermented group (50%DDGS + 50%MGM)
3	Fermented group (50%DDGS + 50%MGM)
4	Unfermented group (62.5%DDGS + 37.5%MGM)
5	Fermented group (62.5%DDGS + 37.5%MGM)

Note: For the control group, only basal diet. For the 50% DDGS + 50% MGM group, wet maize DDGS and air-dried MGM were uniformly mixed at a 50%:50% dry matter ratio. And for the 62.5%DDGS + 37.5% MGM group, wet maize DDGS and dry-based MGM were uniformly mixed at a 62.5%:37.5% dry matter ratio. The mixture was added with 75 mg/kg of cellulase and 97 mg/kg of the X1 enzyme, with a fermentation duration of 82 h, inoculated with 5% lactic acid bacteria, and fermented at 37 °C with 55% moisture content for 82 h. After fermentation, it was air-dried for subsequent use. The fermented group (50% DDGS + 50% MGM) actually refers to the 70% basal diet + 30% fermented (50% DDGS + 50% MGM) group. For the 62.5% DDGS + 37.5% MGM group, wet maize DDGS and dry-based MGM were uniformly mixed at a 62.5:37.5 dry matter ratio. After fermentation, it was air-dried for subsequent use. The fermented group (62.5% DDGS + 37.5% MGM) actually refers to the 70% basal diet + 30% fermented (62.5% DDGS + 37.5% MGM) group.

**Table 3:** Composition and nutritional level of basal diet (air-dried basis) %.

Items	22–42 d
Maize	53.09
Soybean meal	33.00
Flour	5.00
Soya-bean oil	5.50
CaHPO_4_	1.65
Limestone	0.90
NaCl	0.30
L-Lys 55%	0.15
DL-Met 99%	0.10
Threonine 98%	0.00
Composite mineral premix ^a^	0.10
Antioxidant	0.03
Choline chloride	0.15
Multidimensional premix ^b^	0.035
Total	100.00
Calculated nutritive value	
ME (Mcal/kg) ^c^	3.151
Crude protein (%) ^c^	19.86
Calcium (%)	0.81
Available phosphorus (%)	0.40
Lysine (%)	1.11
Methionine (%)	0.41
Threonine (%)	0.78
Arginine (%)	1.36
Valine (%)	0.90
Methionine + Cystine (%)	0.65

Note: ^a^ compound mineral premix per kilogram of full price material provided: Fe, 40 mg; Cu, 16 mg; Zn, 100 mg; Mn, 120 mg; I, 1.25 mg; Se, 0.30 mg. ^b^ available in full price per kg of multidimensional premix: VA, 12,000 IU; VD3, 4500 IU; VE, 24 IU; VK3, 3 mg; VB1, 3 mg; VB2, 9.6 mg; VB6, 3 mg; VB12, 0.018 mg; pantothenic acid, 15 mg; nicotinic acid, 39 mg; folic acid, 1.5 mg; biotin, 0.15 mg. ^c^ the crude protein and ME were determined values (air-dry basis). The experimental diets for the other groups were formulated by iso-proportionally substituting 30% of the basal diet with the solid-state fermented product. And the nutritional composition of the experimental diets was as follows: Unfermented 50% DDGS + 50% MGM diet: 93.08% DM, 18.37 MJ/kg gross energy, 19.25% crude protein, 20.07% neutral detergent fiber (NDF), and 6.7% acid detergent fiber (ADF). Unfermented 62.5% DDGS + 37.5% MGM diet: 93.65% DM, 18.45 MJ/kg gross energy, 19.40% crude protein, 22.70% NDF, and 11.5% ADF. Fermented 50% DDGS + 50% MGM diet: 92.63% DM, 18.16 MJ/kg gross energy, 19.18% crude protein, 19.05% NDF, and 7.2% ADF. Fermented 62.5% DDGS + 37.5% MGM diet: 92.13% DM, 18.23 MJ/kg gross energy, 19.56% crude protein, 18.64% NDF, and 14.4% ADF. All parameters, including DM, gross energy, crude protein, NDF, and ADF, were based on analytically determined values.

**Table 4:** Determination of mobile phase gradient of monosaccharides by liquid chromatography.

Time [min]	A[%]	B[%]	Flow [mL/min]	Max. Pressure Limit [bar]
0.00	100.0	0.0	0.700	400.00
3.00	85.0	15.0	0.700	-----
22.00	82.0	18.0	0.700	-----
24.50	0.0	100.0	0.700	-----
26.00	0.0	100.0	0.700	-----
26.10	85.0	15.0	0.700	-----
30.00	85.0	15.0	0.700	-----

Note: Mobile phase A and B typically represent the two primary solvents constituting the gradient elution program. Mobile phase A was 0.1% (*v*/*v*) formic acid in water; mobile phase B was 0.1% (*v*/*v*) formic acid in pure acetonitrile.

**Table 5:** Economic benefit analysis of broilers.

Item	Maize	Soybean Meal	Fermented Feed (Including Cost of Enzymes)	Fermented Feed Manpower + Equipment Depreciation and Other Costs	Price Difference
Price RMB/ton	2400	2908	1804.33	20	-
Compound feed replacement ratio	2.9%	2.1%	5%		-
Total	69.60	61.07	90.22	20	20.45

Note: For SSF of the combination comprising wet maize DDGS and air-dried MGM, the required enzyme dosages were 75.0 mg/kg of cellulase and 97.0 mg/kg of X1 enzyme. The enzymatic cost per metric ton of fermented feed was calculated at 4.33 RMB/ton. The total production cost of the fermented feed (including maize and soybean meal) reached 1800.00 RMB/ton. All feed ingredient costs were based on 2024 Chinese feed market prices (https://www.feedtrade.com.cn/sbm/index.html, accessed on 1 January 2024), with equipment and labor expenses factored in according to practical operational conditions.

**Table 6:** Optimizing fermentation time.

Item	Reduced Sugar Content mg/g	DMS %
24 h	177.27 ^b^	48.22 ^b^
48 h	200.26 ^a^	48.77 ^b^
72 h	175.85 ^b^	47.75 ^b^
96 h	120.51 ^c^	52.53 ^a^
SEM	8.193	0.847
*p*-value	<0.001	0.002

Different lowercase letters within the same row indicate statistically significant differences between groups (*p* < 0.05), while groups sharing the same letter or without superscript letters show no significant differences (*p* > 0.05). The same notation applies to subsequent tables. DMS, dry matter solubility.

**Table 7:** Variance analysis of regression equation.

Source	Sum of Squares	df	Mean Square	F-Value	*p*-Value	
Model	185.10	9	20.57	7.39	0.0076	significant
A—Cellulase addition	6.11	1	6.11	2.20	0.1819	
B—X1 addition amount	103.90	1	103.90	37.35	0.0005	
C—fermentation time	50.30	1	50.30	18.08	0.0038	
AB	0.5852	1	0.5852	0.2104	0.6604	
AC	0.1024	1	0.1024	0.0368	0.8533	
BC	2.56	1	2.56	0.9204	0.3693	
A^2^	0.1028	1	0.1028	0.0370	0.8530	
B^2^	15.62	1	15.62	5.62	0.0496	
C^2^	4.54	1	4.54	1.63	0.2420	
Residual	19.47	7	2.78			
Lack of Fit	7.55	3	2.52	0.8437	0.5371	not significant
Pure Error	11.92	4	2.98			
Cor Total	204.57	16				

**Table 8:** Model reliability analysis.

Std. Dev.	1.67	R^2^	0.9048
Mean	44.88	Adjusted R^2^	0.7825
C. V.%	3.72	Predicted R^2^	0.7188
		Adeq Precision	9.8926

**Table 9:** Effects of SSF of maize DDGS-MGM on metabolic energy of broilers.

Item	Total Feed Intake (g)	GE (MJ/kg)	N in Feed (%)	N in Excreta (%)	AME (MJ/kg)	AMEn (MJ/kg)	AME/GE%	AMEn/GE%
Control group	437.17 ^a^	18.01	18.95	28.06	13.99 ^a^	13.69 ^a^	0.78 ^a^	0.76 ^a^
Unfermented group (50%DDGS + 50%MGM)	395.12 ^b^	18.38	19.86	24.28	11.87 ^c^	11.64 ^c^	0.65 ^d^	0.63 ^d^
Fermented group (50%DDGS + 50%MGM)	412.51 ^ab^	18.17	19.79	27.33	12.69 ^c^	12.46 ^b^	0.70 ^b^	0.69 ^b^
Unfermented group (62.5%DDGS + 37.5%MGM)	420.97 ^ab^	18.46	20.01	26.79	12.63 ^bc^	12.19 ^b^	0.67 ^c^	0.66 ^c^
Fermented group (62.5%DDGS + 37.5%MGM)	411.63 ^ab^	18.24	20.17	28.89	12.84 ^b^	12.62 ^b^	0.70 ^b^	0.69 ^b^
SEM	10.215	-			0.388	0.197	0.011	0.011
*p*-value	0.011	-			<0.001	<0.001	<0.001	<0.001

Diverse superscripts ^(a–d)^ indicate significant group differences (*p* < 0.05). GE, Gross energy; AME, apparent metabolizable energy; AMEn, nitrogen corrected apparent metabolizable energy. The unfermented (50% DDGS + 50% MGM) group had the following composition: DM 91.05%, crude protein 23.13%, NDF 39.08%, ADF 11.80%, and gross energy of 18.79 MJ/kg. The unfermented (62.50% DDGS + 37.50% MGM) group showed the following: DM 90.69%, crude protein 22.09%, NDF 37.14%, ADF 11.53%, and gross energy of 18.99 MJ/kg. For the fermented (50% DDGS + 50% MGM) group, the values were as follows: DM 89.83%, crude protein 21.76%, NDF 24.09%, ADF 7.10%, and gross energy of 18.40 MJ/kg. Lastly, the fermented (62.50% DDGS + 37.50% MGM) group resulted in DM 88.74%, crude protein 22.98%, NDF 25.25%, ADF 7.50%, and gross energy of 18.43 MJ/kg.

**Table 10:** Determination of metabolic energy of samples to be tested.

Item	AME (MJ/kg)	AMEn (MJ/kg)
Unfermented group (50%DDGS + 50%MGM)	6.92 ^b^	6.69 ^b^
Fermented group (50%DDGS + 50%MGM)	9.66 ^a^	9.42 ^a^
Unfermented group (62.5%DDGS + 37.5%MGM)	8.74 ^a^	8.51 ^a^
Fermented group (62.5%DDGS + 37.5%MGM)	10.14 ^a^	9.93 ^a^
SEM	0.243	0.230
*p*-value	0.005	0.005

Diverse superscripts ^(a,b)^ indicate significant group differences (*p* < 0.05). The analyzed nutritional values of the fermented diet (50%DDGS + 50%MGM) were as follows: DM was 89.83%, crude protein content was 21.16%, metabolizable energy was 9.66 MJ/kg, and NDF and ADF contents were 24.09% and 7.09%, respectively. And the analyzed nutritional values of the fermented diet (62.5%DDGS + 37.5%MGM) were as follows: DM was 88.74%, crude protein content was 22.37%, metabolizable energy was 10.14 MJ/kg, and NDF and ADF contents were 25.25% and 7.50%, respectively.

## Data Availability

The data used to support the findings of this study are available from the corresponding author upon request.
